# In Vitro Cytotoxicity of Three Dimethacrylate-Based Photopolymer Resins for 3D-Printed Dental Restorations: A Qualitative Morphological Screening on Human Fibroblasts

**DOI:** 10.3390/jfb17080421

**Published:** 2026-08-20

**Authors:** Teodora-Alina Tincea, Alexia-Ecaterina Cârstea, Vlad-Gabriel Vasilescu, Andreia Cucuruz, Adela Banciu, Adrian-Ionuț Nicoară, Claudia-Gabriela Mateiaș, Ana-Maria Cristina Țâncu, Silviu-Mirel Pițuru, Marina Imre, Lucian-Toma Ciocan

**Affiliations:** 1 Faculty of Medical Engineering, National University of Science and Technology Politehnica Bucharest, 1-7 Gh. Polizu Street, District 1, 011061 Bucharest, Romania; teodora.tincea@stud.fim.upb.ro (T.-A.T.); adela.banciu@upb.ro (A.B.); adrian.nicoara@upb.ro (A.-I.N.); 2 Discipline of Dental Prosthesis Technology, Faculty of Dentistry, “Carol Davila” University of Medicine and Pharmacy, 37 Dionisie Lupu Street, District 2, 020021 Bucharest, Romania; alexia-ecaterina.carstea2023@stud.umfcd.ro (A.-E.C.); lucian.ciocan@umfcd.ro (L.-T.C.); 3 Department of Prosthodontics, Faculty of Dentistry, “Carol Davila” University of Medicine and Pharmacy, 37 Dionisie Lupu Street, District 2, 020021 Bucharest, Romania; claudia.mateias@umfcd.ro (C.-G.M.); anamaria.tancu@umfcd.ro (A.-M.C.Ț.); marina.imre@umfcd.ro (M.I.); 4 Department of Professional Organization and Medical Legislation-Malpractice, “Carol Davila” University of Medicine and Pharmacy, 37 Dionisie Lupu Street, District 2, 020021 Bucharest, Romania; silviu.pituru@umfcd.ro

**Keywords:** additive manufacturing, 3D printing, vat photopolymerization, dimethacrylate resins, cytotoxicity, biocompatibility, ISO 10993-5, residual monomers, human fibroblasts

## Abstract

The rapid adoption of vat photopolymerization (3D printing) in restorative and prosthetic dentistry has outpaced the independent biological characterization of the dimethacrylate resins on which it relies, even though incompletely converted networks can release residual monomers that are cytotoxic to the adjacent oral tissues. This study reports a qualitative in vitro cytotoxicity screening of three commercial photopolymer resins optimized for additive manufacturing: a general-purpose resin (ANYCUBIC) and two resins with a declared dental indication (TEMP PRINT and V-PRINT) performed according to the morphological evaluation described in ISO 10993-5. Aqueous extracts were prepared following ISO 10993-12 and applied to normal human fibroblasts (BJ line, ATCC CRL-2522) at 100% and 50% concentration for 24 h; morphological reactivity was graded on the standardized 0–4 scale by inverted light microscopy against an unexposed control, and supported by a semi-quantitative visual estimate of the proportion of affected cells. All three materials produced a concentration-dependent response. TEMP PRINT retained the best cellular compatibility (grade 1 at 50%, grade 2 at 100%), V-PRINT was intermediate, and ANYCUBIC showed the most pronounced reactivity (approaching grade 3 at 100%), giving an estimated cytotoxicity order of ANYCUBIC > V-PRINT > TEMP PRINT. As a qualitative morphological screening, the findings indicate that the dental-grade resins elicited a milder fibroblast response than the general-purpose photopolymer and support the recommendation that general-purpose resins should not be substituted for certified materials in intraoral use. Confirmation by quantitative viability assays with independent replicates is required before firm conclusions are drawn. In line with the qualitative nature of ISO 10993-5 morphological screening, the reported proportions of morphologically affected cells represent visual scoring rather than direct measurements of cell viability, and the study is therefore presented as an initial biological screening.

## 1. Introduction

Contemporary restorative and prosthetic dentistry is undergoing a rapid transition toward fully digital workflows, in which computer-aided design and computer-aided manufacturing (CAD/CAM), and increasingly, additive manufacturing (AM, or three-dimensional [3D] printing), are reshaping clinical and laboratory procedures [1,2]. Among the additive routes, vat photopolymerization, principally stereolithography (SLA) and digital light processing (DLP), has become the dominant approach for polymer-based dental devices [3,4], and photopolymerizable dimethacrylate resins are now used for provisional and definitive restorations, occlusal splints, removable-prosthesis bases, and study models [5]. Because these devices are in direct and often prolonged contact with the oral mucosa, their clinical acceptability depends not only on the fabrication accuracy, but critically on their biological safety toward the surrounding soft tissues [6,7].

Dimethacrylate networks are formed by the free-radical photopolymerization of monomers such as bisphenol A-glycidyl methacrylate (Bis-GMA), urethane dimethacrylate (UDMA), and triethylene glycol dimethacrylate (TEGDMA), whose relative proportions govern the degree of conversion, and ultimately, biological performance [8,9]. Photopolymerization is inherently incomplete: as the network vitrifies during curing, chain mobility becomes restricted and a fraction of the monomer remains unreacted and physically entrapped within the matrix [10]. In vat-photopolymerized devices, the final degree of conversion, and hence the residual-monomer burden, is strongly dependent on the post-processing protocol, particularly the washing step and the intensity, duration and temperature of post-curing [11,12,13].

Once eluted into saliva or crevicular fluid, residual monomers can diffuse into the adjacent tissues, cross cell membranes and trigger oxidative stress, glutathione depletion, and ultimately, apoptotic or necrotic cell death [14,15]; photoinitiators and co-initiators can further contribute to oxidative and mitochondrial stress [16]. Systematic and meta-analytical evidence confirms that measurable quantities of Bis-GMA, TEGDMA, and UDMA are released from resin-based dental materials including 3D-printed composites [17,18]. Consistent with this, adequate washing and effective post-curing have been shown to lower the extractable fraction, and correspondingly, the cytotoxicity of printed resins [19,20,21,22].

Restorations and appliances 3D-printed from dimethacrylate resins fall within the scope of Regulation (EU) 2017/745 on medical devices (MDR); under Annex VIII, provisional intraoral restorations are typically classified as Class IIa devices, and any device of this class contacting the oral mucosa must undergo biological evaluation in accordance with ISO 10993-5 [23,24]. A recurring practical concern is the substitution of inexpensive general-purpose photopolymers for certified dental-grade materials, whose biological equivalence is often assumed rather than demonstrated. Direct comparisons have shown that 3D-printed resins can exhibit greater cytotoxicity toward L929 cells and human gingival fibroblasts than milled or conventional counterparts when residual monomer is not adequately removed [25,26,27]. Because gingival and periodontal fibroblasts are the resident majority cell population of the soft tissues first exposed to leachables from intraoral restorations, they constitute a clinically relevant model for such screening [28].

The aim of the present study was to provide a qualitative in vitro cytotoxicity screening of three commercially available photopolymer resins for additive manufacturing, one general-purpose resin (ANYCUBIC) and two resins carrying a declared dental indication (TEMP PRINT and V-PRINT), using the morphological evaluation described in ISO 10993-5. Aqueous extracts prepared according to ISO 10993-12 were applied to normal human fibroblasts at two concentrations, and morphological reactivity was graded on the standardized 0–4 scale in order to compare the biological response elicited by a general-purpose photopolymer with that of certified dental-grade resins.

According to the manufacturers’ publicly available safety data sheets and technical documentation, the three materials share (meth)acrylate photopolymer chemistry but differ in formulation. ANYCUBIC, a general-purpose UV resin, is declared to contain a polyurethane (urethane) acrylate oligomer (30–60%; CAS 82116-59-4), an acrylate monomer (10–40%; CAS 29590-42-9), and a photoinitiator (2–5%). TEMP PRINT (GC Temp PRINT) is a light-curing, methacrylate-based composite for temporary restorations, described as a urethane dimethacrylate (UDMA)-based system containing inorganic filler and a photoinitiator. V-PRINT (VOCO V-Print c&b temp) is a light-curing, methacrylate-based composite printing material for temporaries, containing dimethacrylate monomers, inorganic fillers, and a photoinitiator.

## 2. Materials and Methods

### 2.1. Study Design

This was a qualitative, comparative in vitro study in which the cytotoxicity of three commercial photopolymer resins: one general-purpose (ANYCUBIC) and two with a declared dental indication (TEMP PRINT and V-PRINT), was screened by morphological evaluation according to ISO 10993-5. For each material, disc-shaped specimens were sterilized and used to prepare aqueous extracts following ISO 10993-12; the extracts were applied to normal human BJ fibroblasts (ATCC CRL-2522) at two concentrations (100% and 50%) and compared with an unexposed control culture. The primary endpoint was the categorical ISO 10993-5 reactivity grade (0–4), supported by a semi-quantitative visual estimate of the proportion of morphologically affected cells. The study was designed as a qualitative morphological screening; quantitative viability assays and inferential statistical analysis were outside its scope and are identified as necessary future work.

### 2.2. Materials

Three commercial photosensitive composite resins optimized for additive-manufacturing technologies were evaluated: ANYCUBIC, a general-purpose resin, and TEMP PRINT and V-PRINT, both having a declared dental indication. The base chemistry of all three materials is a complex dimethacrylate polymer matrix; monomers frequently used in such systems, such as Bis-GMA, UDMA, and TEGDMA, form a strongly cross-linked three-dimensional network following free-radical photopolymerization under UV light. Specimens for the present assay were produced as discs by vat photopolymerization followed by the manufacturers’ standard post-processing procedure. A detailed presentation of the declared constituents of each resin, together with the distinction between the general-purpose product and the two dental-grade materials, is provided in the Introduction. All three resins were used exactly as supplied by the manufacturers, without any modification.

#### Specimen Fabrication and Post-Processing

Disc-shaped specimens, 10 mm in diameter and 1.5 mm in thickness, were designed in the open-source modeling software Blender (v3.3 LTS; Blender Foundation, Amsterdam, The Netherlands) and produced by vat photopolymerization on a Planmeca Creo C5 medical-grade LCD printer (Planmeca Oy, Helsinki, Finland) with a light source emitting at 405 nm. Three discs were printed for each material. Once the build was complete, the specimens were detached from the platform and post-processed under a standardized protocol: the uncured surface layer was first dissolved by immersion in 2-propanol (>90%), after which the discs were UV post-cured to increase the degree of monomer conversion and reduce residual porosity, in each case using the post-curing time and UV intensity specified by the respective manufacturer. Solvent cleaning was performed with 2-propanol. The disc geometry (10 × 1.5 mm) corresponded to a surface-area-to-volume ratio of approximately 2 cm^2^ per mL of extraction medium, consistent with the conditions recommended by ISO 10993-12.

### 2.3. Cell Model

Normal human fibroblasts of the BJ line (ATCC CRL-2522) were used as the cell model. Fibroblasts are mesenchymal cells that constitute the resident majority population of the gingival connective tissue and periodontal ligament; they synthesize and remodel the extracellular matrix (collagen, elastin, glycosaminoglycans) and are directly responsible for wound healing, tissue regeneration, and the maintenance of the biological seal around implanted or restorative dental materials. As the predominant and first-exposed cell type in the gingival connective tissue, they confer high clinical relevance on the assay. Cells were maintained in Eagle’s minimum essential medium supplemented with 10% fetal bovine serum, in a humidified incubator at 37 °C and 5% CO_2_, which maintained the bicarbonate-buffered medium at a physiological pH of approximately 7.2 to 7.4.

### 2.4. Specimen Conditioning and Sterilization

Cross-contamination with prokaryotes or fungi had to be eliminated, because the nutrient-rich culture medium would otherwise allow microbial proliferation that lyses fibroblasts independently of the tested material and generates false-positive results. To prevent this artifact, a physical sterilization protocol was applied under a laminar-flow microbiological hood. Each specimen was exposed to UVC radiation (approximately 254 nm) on both faces for 20 min per face. UVC induces pyrimidine dimer formation in microbial DNA, irreversibly blocking transcription and replication. Compared with chemical or thermal methods such as autoclaving at 121 °C, UV exposure is preferred for polymers because it induces neither thermal degradation, hydrolysis, nor structural distortion; for dimethacrylate resins, the additional photon flux further completes surface photopolymerization and stabilizes the oxygen-inhibited layer.

### 2.5. Preparation of Standardized Extracts

The toxicity of leachable species was assessed indirectly through aqueous extracts, following the parameters of ISO 10993-12 [29]. Sterilized specimens were individually immersed in vials containing 1 mL of complete culture medium and extracted for 72 h. Because monomer release from dense three-dimensional networks is a slow diffusion-controlled process governed by matrix porosity, cross-link density, and the molecular size of the leachable species, the 72 h extraction was intended to approach equilibrium between material and vehicle and to drive into solution the unreacted photoinitiators, additives, stabilizers, and free monomers, producing a conservative worst-case toxicological profile.

### 2.6. Cell Exposure and Simulation of Salivary Clearance

Because the continuous salivary flow in the oral cavity exerts a marked washing and dilution (clearance) effect, extracts were tested on subconfluent BJ fibroblasts at two levels. The 100% group represented the absolute concentration, mimicking the intimate, stagnant contact of deep interdental areas or the abutment–crown interface where salivary washing is minimal. The 50% group was prepared by diluting the original extract 1:1 (*v*/*v*) with fresh culture medium, mimicking the protective effect of oral fluids and providing a dose–response effect. Once inoculated, cultures were incubated for a further 24 h, an interval established in cellular toxicology for observing the onset of acute effects on the cell cycle and intracellular energy metabolism. The 1:1 (50%) dilution was selected as a standard second test concentration to establish a concentration–response relationship, consistent with the common use of serial or two-point extract dilutions in ISO 10993-5 cytotoxicity testing [24]; the 50% level was not intended to represent a precise physiological clearance value but to provide a diluted-exposure condition complementary to the undiluted (100%) extract.

### 2.7. Morphological Evaluation and Grading

Morphological analysis at the monolayer level was performed using a Leica DMi8 inverted research microscope (Leica Microsystems, Wetzlar, Germany). [30]. The inverted configuration allows for the examination of Petri-dish cultures through their transparent optical-plastic base without exposing or contaminating the medium layer in which the cells are immersed. Micrographs were examined for clear signs of cellular distress: fibroblasts exposed to toxic agents depolymerize the actin cytoskeleton, which leads to loss of the fusiform shape, retraction of filopodia and cell rounding; the focal-adhesion complexes anchoring the cell are then disrupted and the cell detaches, a stage that precedes membrane lysis. The process was graded according to the ISO 10993-5 scale [24], assigning a reactivity grade from 0 (no reaction, excellent morphology) to 4 (severe reactivity, complete destruction of the monolayer); the grade was supported by a semi-quantitative visual estimate of the proportion of morphologically affected cells, used only as an aid to grading and not as a measured viability value. The grading criteria are summarized in Table 1. Cultures were examined live and unstained by inverted transmitted-light (phase-contrast) microscopy so that the same cultures could be followed without fixation or processing artifacts, which is a configuration suited to the direct morphological evaluation of adherent monolayers under ISO 10993-5. Histochemical methods such as Masson’s trichrome are designed for fixed, sectioned tissue and for the visualization of collagen and connective-tissue architecture, and are therefore not applicable to the real-time assessment of a living fibroblast monolayer; for this reason, such stains were not used. For each condition, a representative field illustrating the typical monolayer appearance was documented, with fields selected to avoid plate edges, air bubbles, and drying artifacts.

## 3. Results

### 3.1. Control Culture

**Figure 1:**
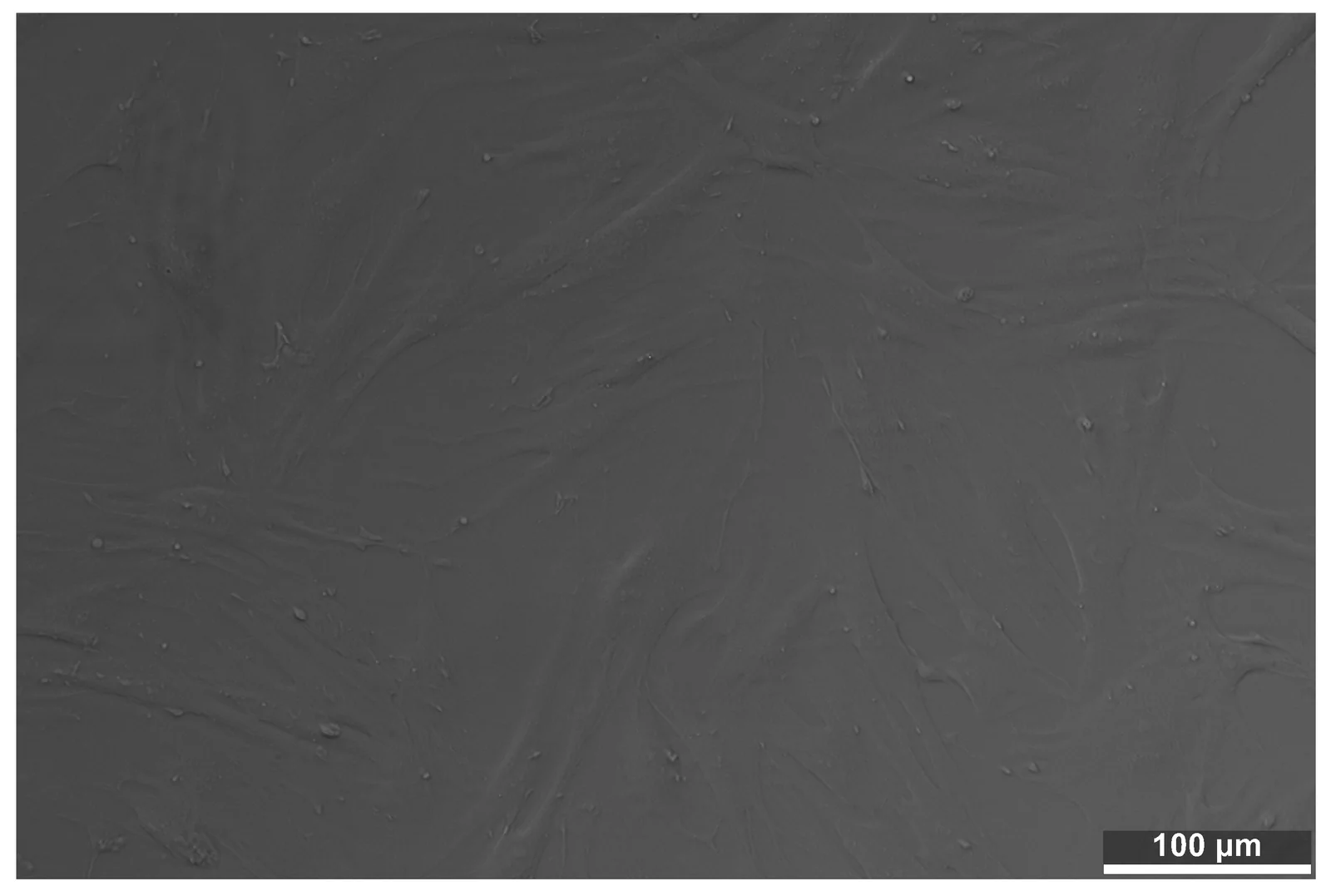
Control BJ fibroblast culture, not exposed to any extract, used as the morphological reference. Confluent monolayer of elongated, fusiform cells with intact cytoplasmic extensions (grade 0). Scale bar, 100 µm.

The control culture, maintained exclusively in complete sterile medium and not exposed to any extract, exhibited optimal development and proliferation. A dense cellular network was observed as a monolayer approaching maximum confluence. Phenotypically, the fibroblasts displayed a healthy morphology characteristic of this primary line: a flattened, strongly elongated (fusiform) shape with complex reticular organization and multiple thin cytoplasmic extensions through which the cells anchored firmly to the substrate and established cell–cell contacts (Figure 1). Applying the ISO 10993-5 grid, the control was classified at grade 0 (no reactivity): no rarefaction or reduction in growth, no cell rounding, no detachment, no stress vacuoles, and no floating cellular debris. This image served as the reference against which all treated samples were interpreted (Table 2).

### 3.2. ANYCUBIC

**Figure 2:**
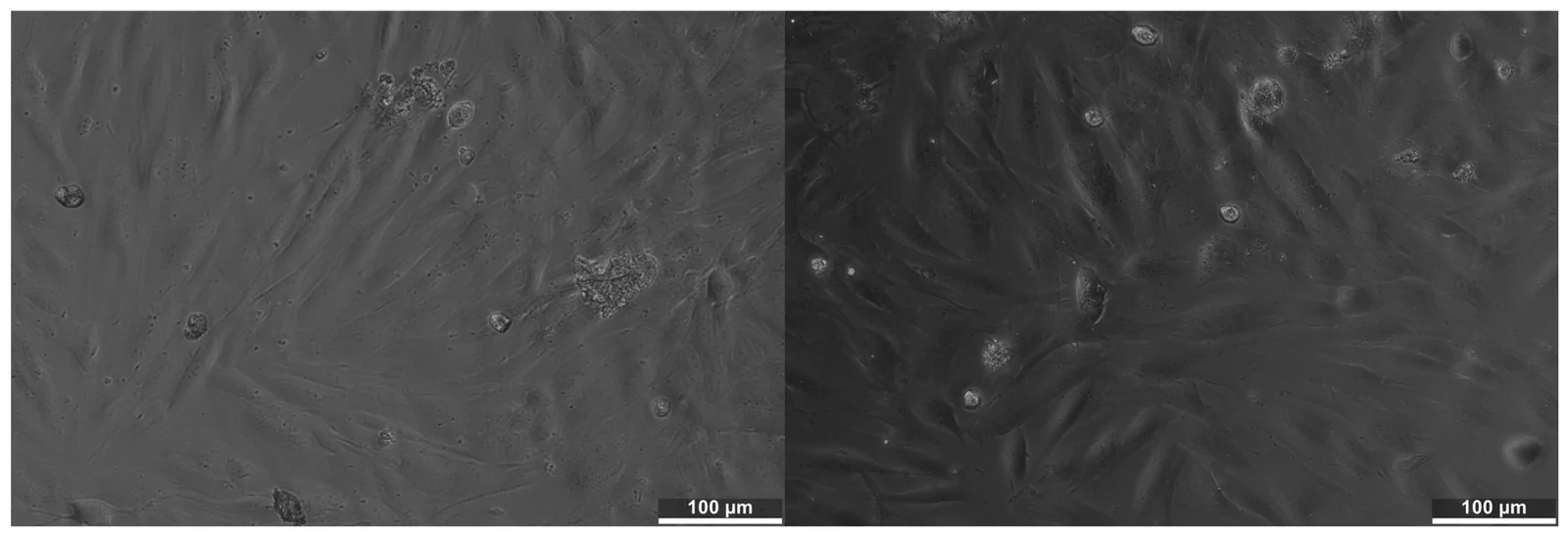
BJ fibroblasts exposed to ANYCUBIC extracts. (**Left**) 50% extract.(**Right**) 100% extract. Progressive rounding, loss of the fusiform morphology, and increased refractile debris are evident with increasing concentration. Scale bar, 100 µm.

In the 50% extract, the culture retained a substantial number of adherent cells, but the network was no longer uniform and several regions showed rounded cells, refractile particles, and aggregates of cellular debris, indicating that cellular stress had already appeared at the diluted concentration. Approximately 20% to 30% of cells appeared morphologically affected, corresponding to a slight-to-mild reactivity between grades 1 and 2. In the 100% extract, the changes were markedly more pronounced: reduced cell density, a greater number of rounded cells, and a more evident loss of monolayer organization, with regions in which the fusiform morphology was partially lost. The estimated proportion of affected cells rose to approximately 45% to 55%, indicating a moderate reactivity approaching grade 3. The transition from 50% to 100% therefore produced the clearest concentration-dependent worsening among the three materials. The probable cause is the release of residual compounds such as unpolymerized monomers, oligomers, photoinitiators, and additives, which at a higher concentration can affect the cell membrane, mitochondrial metabolism, and cytoskeletal organization (Figure 2).

### 3.3. TEMPPRINT

**Figure 3:**
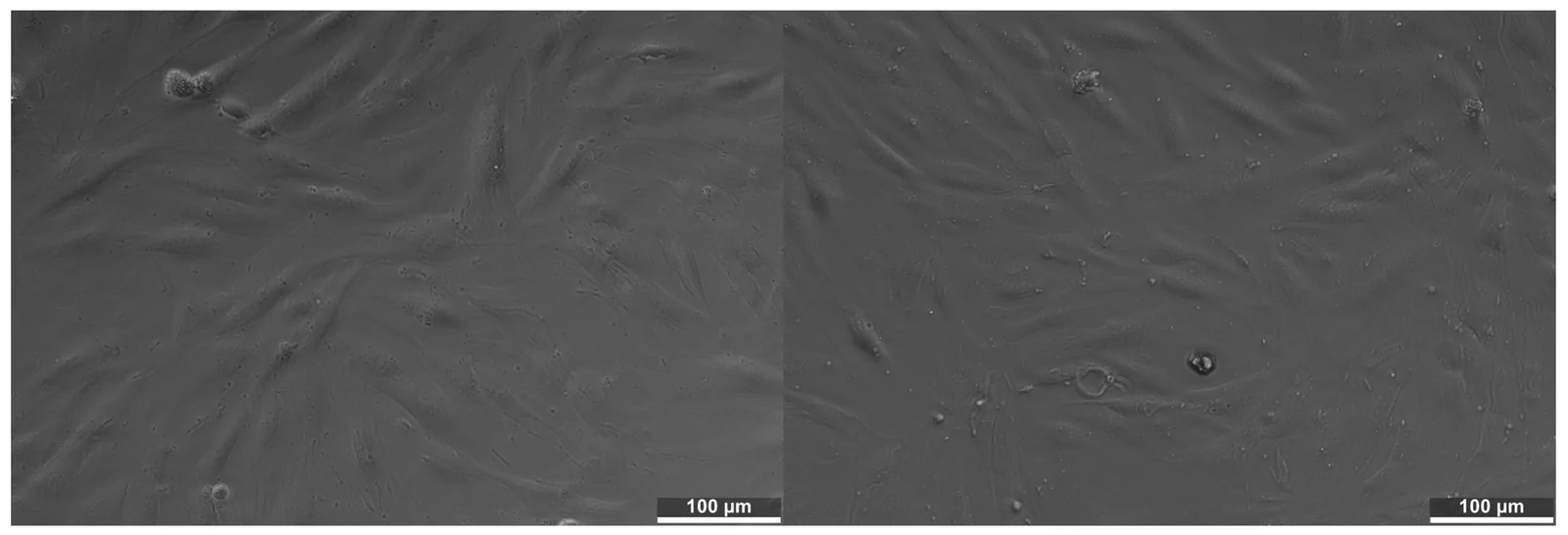
BJ fibroblasts exposed to TEMP PRINT extracts. (**Left**) 50% extract. (**Right**) 100% extract. The monolayer is largely preserved at both concentrations, with only isolated rounded cells. Scale bar, 100 µm.

In the 50% extract, the overall appearance of the culture was well-preserved: the majority of cells retained their elongated, fusiform shape and good adhesion, with only minor signs of stress such as isolated rounded cells and a few regions of slightly reduced density. Approximately 10% to 15% of cells appeared affected, corresponding to grade 1 (slight reactivity). In the 100% extract, the effects were more visible than at 50% but the monolayer remained largely conserved; cytoplasmic extensions were still evident and the cell distribution remained close to that of the control. Approximately 20% to 30% of cells appeared affected, indicating a slight-to-mild reactivity close to grade 2. Of the three materials, TEMP PRINT showed the smallest increase in effect between 50% and 100%, consistent with a more efficient polymerization, a lower release of residual monomers, or a more stable chemical composition (Figure 3).

### 3.4. V-PRINT

**Figure 4:**
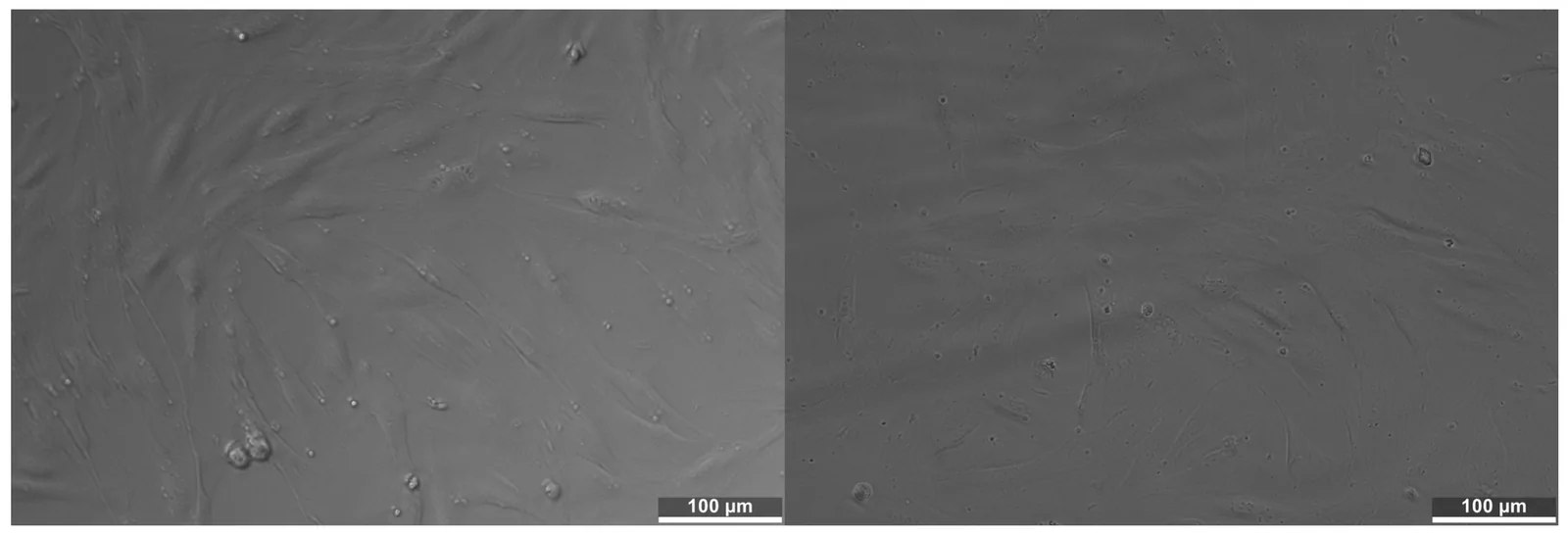
BJ fibroblasts exposed to V-PRINT extracts. (**Left**) 50% extract. (**Right**) 100% extract. Intermediate response, with a viable population at 50% and clearer rarefaction and cell retraction at 100%. Scale bar, 100 µm.

In the 50% extract, the culture remained viable, with numerous adherent, elongated cells; however, the density appeared slightly reduced in some regions and the number of rounded cells was higher than in the control, corresponding to a slight-to-mild reactivity (grades 1 to 2, approximately 15% to 25% of cells affected). In the 100% extract, the changes were more evident: the culture appeared more rarefied, with clearer signs of cell retraction and rounding and more refractile elements. Approximately 30% to 40% of cells appeared affected, indicating a moderate reactivity classifiable as grade 2. V-PRINT thus occupied an intermediate biological position: less aggressive than ANYCUBIC at 100% but more reactive than TEMP PRINT (Figure 4).

### 3.5. Effect of Extract Concentration

In all three cases, the undiluted (100%) extract produced more pronounced changes than the diluted (50%) extract, consistent with a dose-dependent release of soluble compounds. On the basis of visible morphological alteration, the estimated cytotoxicity order was ANYCUBIC > V-PRINT > TEMP PRINT. Throughout the Results section, the percentages of morphologically affected cells are reported as visual, semi-quantitative scores obtained from inverted-microscopy images and do not represent measured cell viability (Table 3).

### 3.6. Summary of ISO 10993-5 Reactivity Grades

To allow a direct, standardized comparison, the morphological observations described above were consolidated into the categorical reactivity grade defined by ISO 10993-5 (Table 4). The unexposed control was graded 0. In the 50% extract, the two dental-grade resins remained within grade 1 (TEMP PRINT) to the grade 1–2 range (V-PRINT), while ANYCUBIC already reached the grade 1–2 range. In the undiluted (100%) extract, TEMP PRINT and V-PRINT did not exceed grade 2, whereas ANYCUBIC approached grade 3. Applying the criterion of the standard, according to which a reactivity grade greater than 2 indicates a cytotoxic effect, the two dental-grade resins remained below this threshold under the conditions tested, while the general-purpose resin approached it at the undiluted concentration. These grades re-express the qualitative morphological evaluation and are not derived from quantitative viability measurements (Table 4 and Table 5).

## 4. Discussion

In the qualitative cytotoxicity assay, cell death is not produced by mechanical factors but by chemical species leached from the material into the extraction medium, principally residual monomers, photoinitiators, polymerization inhibitors, pigments, additives, and matrix-degradation products [16]. An insufficiently polymerized material releases a larger quantity of unconverted monomer, which can cross the cell membrane and disrupt cellular metabolism, while photoinitiators and co-initiators contribute to oxidative stress and impaired mitochondrial function [10,31]. As the actin cytoskeleton reorganizes and focal-adhesion proteins are compromised, cells lose their fusiform shape, round up, detach, and may die, which is why loss of adhesion is among the most informative morphological signs in this assay. The observed order (ANYCUBIC > V-PRINT > TEMP PRINT) and the consistent dose dependence are congruent with this mechanism and with the wider finding that more completely converted, more stable networks release fewer cytotoxic species [7,14,17,18,32].

The results are consistent with recent reports on vat-photopolymerized dental resins. Direct comparisons have shown that 3D-printed resins can be more cytotoxic toward L929 cells and human gingival fibroblasts than milled or conventional counterparts when residual monomer is not adequately removed [25,26,27], whereas well-processed printed materials can support good fibroblast morphology and adhesion [28]. Importantly, the biological response reflects not only intrinsic chemistry, but also processing: adequate washing and a more effective post-curing protocol reduce the residual-monomer burden, and hence cytotoxicity [19,20,21,22]. Within this framework, the milder fibroblast reactivity of the two dental-grade resins, and the more pronounced response of the general-purpose photopolymer, are best interpreted as the combined result of composition and post-processing rather than of chemistry alone [6]. The finding provides qualitative experimental support for the regulatory expectation that general-purpose photopolymers should not be substituted for certified dental-grade resins in intraoral use.

The concentration-dependent pattern observed for all three materials is central to the interpretation of these results. Testing each extract at both 100% and 50% was intended to reproduce, in a simplified way, the concentration gradient that leachable species encounter in vivo: an almost undiluted exposure in stagnant, poorly cleared sites such as deep interdental spaces or the abutment–crown interface, and a diluted exposure where salivary flow provides continuous washing. The consistent worsening of morphological reactivity from 50% to 100% indicates that the observed effects scale with the quantity of soluble compounds reaching the cells, which is the expected behavior for a diffusion-controlled release of residual monomers and additives rather than for a fixed, all-or-nothing toxic threshold [14,15,17]. Clinically, this suggests that the biological risk associated with a given resin is not constant but depends on the local balance between elution and salivary clearance, and that sites with limited salivary access warrant particular attention.

The finding that the general-purpose resin (ANYCUBIC) elicited the most pronounced fibroblast reactivity, while the two resins with a declared dental indication remained closer to the control, is consistent with the way that these materials are formulated and certified. Dental-grade resins are developed and validated for intraoral contact and are supported by defined post-processing protocols, whereas general-purpose photopolymers are optimized for dimensional accuracy and surface finish rather than for biological contact, and typically carry no such validation [22,23,24]. A higher unreacted-monomer or photoinitiator burden in an uncertified material provides a plausible explanation for its stronger cytotoxic signal, and is in line with reports that printed resins can display greater cytotoxicity than milled or conventional counterparts when residual species are not adequately removed [25,26]. These considerations reinforce the regulatory expectation, under the medical-device framework, that any material contacting the oral mucosa should undergo formal biological evaluation before clinical use [23,24].

The choice of normal human fibroblasts as the test model strengthens the clinical relevance of the screening. Fibroblasts constitute the resident majority population of the gingival connective tissue and are among the first cells exposed to species leached from an intraoral restoration, so their response is a meaningful surrogate for the soft-tissue reaction at the restoration interface [6,27]. The morphological endpoints used here, namely loss of the fusiform shape, retraction of cytoplasmic extensions, cell rounding, and detachment, are recognized early indicators of cytotoxic stress: because anchorage-dependent cells rely on intact focal adhesions for survival, loss of adhesion typically precedes membrane lysis and is therefore an informative, if qualitative, marker of biological injury [7]. Reassuringly, well-processed printed materials have been shown to support good fibroblast morphology and adhesion, which indicates that the reactivity observed here reflects material- and processing-dependent differences rather than an intrinsic incompatibility of printed resins as a class [28].

Taken together, these observations point to post-processing as a decisive and modifiable determinant of biological safety. The degree of conversion attained after printing, and hence the quantity of extractable monomer, is strongly governed by the washing step and by the intensity, duration, and temperature of post-curing, and improvements in these parameters have been shown to lower both the residual-monomer content and the resulting cytotoxicity [19,20,21]. The milder response of the dental-grade resins is therefore best interpreted as the combined outcome of a more suitable formulation and a better-defined post-processing route, rather than of chemistry alone [10,16]. This interpretation is consistent with the broader literature on resin biocompatibility, in which more completely converted and more stable networks are repeatedly associated with a reduced release of cytotoxic species and improved cellular tolerance [31,32,33].

Finally, while the present screening was deliberately confined to a qualitative biological endpoint, a complete assessment of clinical suitability will ultimately require the biological behavior reported here to be integrated, in future work, with the mechanical durability of these materials under clinically relevant conditions. Static strength alone is not sufficient, since fatigue, wear, and hydrothermal aging can substantially reduce the performance of printed restorative and splint resins over time [34,35,36,37]. A combined evaluation, in which quantitative viability assays and mechanical testing with adequate replication are performed on the same materials and processing conditions, would allow the qualitative trends observed here to be confirmed and placed in a fully quantitative, clinically actionable framework.

### Limitations

Several limitations must be stated explicitly and temper the strength of the conclusions. First, the study is a qualitative morphological screening: the primary endpoint is the categorical ISO 10993-5 reactivity grade, supported by semi-quantitative visual estimates of the proportion of morphologically affected cells. A rounded cell may be affected, detached, or simply dividing, so the correct term is morphologically affected cell rather than confirmed dead cell, and the estimated proportions must not be read as measured viability. Definitive quantification would require assays such as trypan blue, MTT, XTT, LDH or resazurin (Alamar Blue), together with automated image segmentation and the expression of viability as a percentage of control across independent replicates that permit standard deviations and inferential statistics. Second, cytotoxicity was assessed indirectly through aqueous extracts; the leachables were not chemically characterized, so the specific species responsible for the observed reactivity were not identified. Third, standardization of the post-curing and washing protocol across all materials would remove processing as a confounding variable in the biological comparison. These points define the necessary next steps toward a fully quantitative evaluation and should be addressed before firm clinical recommendations are made. In particular, the degree of conversion of the printed specimens was not measured, and the composition of the eluates was not determined by chromatographic or mass-spectrometric methods; the mechanistic interpretation in terms of residual-monomer and additive release is therefore inferential and should be confirmed by such analyses. Moreover, although three specimens per material were prepared, the morphological evaluation was qualitative and a single representative field was documented per condition, so no quantitative measures of variability or inferential statistics were derived, and adequately powered, replicated, and quantitative assays are required to confirm the trends reported here.

## 5. Conclusions

Within the constraints of a qualitative in vitro morphological screening, this ISO 10993-5 evaluation of three dimethacrylate-based photopolymer resins supports the following conclusions:

- Qualitative morphological evaluation of BJ human fibroblasts revealed a concentration-dependent cytotoxic response for all three materials, with the undiluted (100%) extract consistently producing more pronounced changes than the 50% extract.
- In the 50% extract, morphology remained largely normal for the dental-grade resins; in the 100% extract, TEMP PRINT retained the best cellular compatibility (grade not exceeding 2), whereas ANYCUBIC showed the most pronounced reactivity (approaching grade 3).
- The estimated cytotoxicity order was ANYCUBIC > V-PRINT > TEMP PRINT, indicating that the two dental-grade resins elicited a milder fibroblast response than the general-purpose photopolymer.
- As a qualitative screening, these findings require confirmation by quantitative viability assays with independent replicates and appropriate statistical analysis before firm clinical recommendations can be made; they nonetheless provide qualitative support for preferring certified dental-grade resins over general-purpose photopolymers in intraoral applications.

## Figures and Tables

**Table 1:** Qualitative cytotoxicity grading system according to ISO 10993-5.

Grade	Reactivity	Description of the Cell-Culture Condition (Morphological Changes)
0	None	Discrete intracytoplasmic granules; no cell rounding, no detachment or lysis; no reduction in cell growth rate (healthy, confluent monolayer).
1	Slight	Not more than 20% of cells are rounded, loosely attached, and without intracytoplasmic granules; occasional morphological changes; slight inhibition of growth.
2	Mild	Not more than 50% of cells are rounded and devoid of intracytoplasmic granules; no extensive lysis; growth inhibition up to 50%.
3	Moderate	Up to 70% of the cell layer contains rounded or lysed cells; layer not completely destroyed but growth inhibition exceeds 50%.
4	Severe	Near-complete or total destruction of the monolayer; cells massively lysed and detached, leaving large empty areas.

**Table 2:** Morphological interpretation of the control culture.

Element Assessed	Observation	Interpretation
Morphology	Elongated, fusiform cells with cytoplasmic extensions	Normal appearance of viable fibroblasts
Adhesion	Cells well attached to the substrate	Stable, healthy culture
Confluence	Good cell density, relatively uniform network	Favorable growth conditions
Cellular debris	Very little, no major aggregation	Absence of significant cell degradation
Estimated grade	Grade 0	Reference sample, no visible cytotoxicity

**Table 3:** Semi-quantitative comparison of the concentration effect on cell morphology (estimated proportion of morphologically affected cells).

Material	Extract 50%	Extract 100%	Increase	Comparative Note
ANYCUBIC	20–30%	45–55%	+20–30 p.p.	Highest cytotoxicity
TEMP PRINT	10–15%	20–30%	+10–15 p.p.	Best cell compatibility
V-PRINT	15–25%	30–40%	+15 p.p.	Intermediate behavior

**Table 4:** Summary of ISO 10993-5 reactivity grades assigned by qualitative morphological evaluation.

Material	Extract 50%	Extract 100%	Exceeds Grade 2
Control (unexposed)	Grade 0	Grade 0	No
TEMP PRINT	Grade 1	Grade 2	No
V-PRINT	Grade 1 to 2	Grade 2	No
ANYCUBIC	Grade 1 to 2	Grade 3 (approaching)	Borderline at 100%

**Table 5:** Qualitative morphological features observed by inverted light microscopy.

Condition	Fusiform Morphology	Cytoplasmic Extensions	Cell Rounding	Detachment/Refractile Debris	Monolayer Confluence
Control	Preserved	Abundant, intact	Absent	Absent	Near-confluent
TEMP PRINT 50%	Largely preserved	Evident	Isolated	Minimal	Good
TEMP PRINT 100%	Mostly preserved	Still evident	Occasional	Slight	Good
V-PRINT 50%	Preserved	Evident	Few	Slight	Slightly reduced
V-PRINT 100%	Partly reduced	Reduced	Frequent	Moderate	Reduced
ANYCUBIC 50%	Partly reduced	Reduced	Frequent	Moderate	Non-uniform
ANYCUBIC 100%	Partly lost	Sparse	Extensive	Marked	Clearly reduced

## Data Availability

The original contributions presented in the study are included in the article; further inquiries can be directed to the corresponding authors.
